# Quantitative analysis of the dominant external factors influencing elite speed Skaters' performance using BP neural network

**DOI:** 10.3389/fspor.2024.1227785

**Published:** 2024-02-09

**Authors:** Zhenlong Yang, Peng Ke, Yiming Zhang, Feng Du, Ping Hong

**Affiliations:** ^1^ School of Transportation Science and Engineering, Beihang University, Beijing, China; ^2^ School of Competitive Sports, Beijing Sports University, Beijing, China

**Keywords:** speed skating, BP neural network, ice rink altitude, ice temperature, race frequency

## Abstract

**Introduction:**

Speed skating, being a popular winter sport, imposes significant demands on elite skaters, necessitating their effective assessment and adaptation to diverse environmental factors to achieve optimal race performance.

**Objective:**

The aim of this study was to conduct a thorough analysis of the predominant external factors influencing the performance of elite speed skaters.

**Methods:**

A total of 403 races, encompassing various race distances and spanning from the 2013 to the 2022 seasons, were examined for eight high-caliber speed skaters from the Chinese national team. We developed a comprehensive analytical framework utilizing an advanced back-propagation (BP) neural neural network model to assess three key factors on race performance: ice rink altitude, ice surface temperature, and race frequency.

**Results:**

Our research indicated that the performance of all skaters improves with higher rink altitudes, particularly in races of 1,000 m and beyond. The ice surface temperature can either enhance or impaire performance and varies in its influences based on skaters' technical characteristics, which had a perceptible or even important influence on races of 1,500 m and beyond, and a negligible influence in the 500 m and 1,000 m races. An increase in race frequency generally contributed to better performance. The influence was relatively minor in the 500 m race, important in the 3,000 m race, and varied among individuals in the 1,000 m and 1,500 m races.

**Conclusion:**

The study results offer crucial guidelines for speed skaters and coaches, aiding in the optimization of their training and competition strategies, ultimately leading to improved competitive performance levels.

## 1 Introduction

Speed skating race performance is influenced by numerous factors. Conducting an in-depth analysis of the patterns of influence of these factors is crucial for enhancing performance and optimizing training strategies. Research has investigated the influence of various objective factors on race times, covering gender, season, competition stage, start position, event importance, number of events per tournament, number of competitors per race, altitude, time qualification, atmospheric pressure, rink type and location, geographic latitude, and oxygen concentration (1–4). Additionally, subjective factors, like athletes' technical abilities involving strength, mass, and motor behavior (6, 7), along with game strategy (8), have demonstrated a notable impact on game performance.

However, certain common and intriguing factors like ice surface temperature and race frequency (referring to the number of races a skater attended in a single season) still lack comprehensive research regarding their impact on athletic performance. For instance, the dispute over ice surface temperature settings between professional athletes and ice technicians at the Beijing Winter Olympics highlighted divergent understandings even among professional bodies about the personalized impact of ice surface temperatures on athletes (9). While existing studies indicate that ice surface temperature affects the coefficient of friction (10), there remains a lack of a more holistic understanding of its impact on performance. On the other hand, some scholars have observed that in 500 m and 1,000 m short track speed skating, the completion times in finals, semi-finals, and quarterfinals are likely to be faster than in preliminary rounds (1). However, this effect seems negligible in 1,500 m races. The potential positive or negative impact of competition frequency on athletes has yet to be explored by researchers.

In addressing the impact of various parameters, traditional mathematical and statistical methods are commonly employed, with regression analysis being a frequently utilized technique. For instance, Muehlbauer et al.'s (11) regression analysis revealed that a shorter time spent in the last 400 meters of a 1,000-m race was correlated with a shorter total race time. Knobbe et al. (12) used linear modeling to integrate exercise sequences, discovering that sustaining high-intensity training three weeks pre-competition notably boosted performance, providing strong backing for future training improvements. Konings and Hettinga (1) used mixed linear models on log-transformed lap and finishing times. They revealed that variables like the competition's skater count, stage, and importance could impact skilled skaters' pacing decisions and, consequently, their overall performance. Ichinose et al. (13) employed a linear mixed model to establish a correlation between standardized final rankings and exposure times. They discovered that skaters hid behind others to avoid air resistance for a long time before the final lap tended to win, showcasing the significance of managing energy expenditure throughout the race. Sun et al. (3) created a multivariate regression model, concluding that latitude and altitude were significant factors affecting performance time differences among various rink locations.

While these methods can derive general patterns, they fall short in individualized characterization of specific athletes due to the strong coupling between multiple parameters. Currently, machine learning-based data mining techniques offer new perspectives. Machine learning (ML) methods, such as Support Vector Machine (SVM), Random Forest (RF), Logistic Regression (LR), K-Nearest Neighbors (KNN), Naive Bayes (NB), and Artificial Neural Networks (ANN), have emerged as powerful tools that can enhance prediction efficiency and accuracy compared to regression analysis (14). ML techniques have gained significant popularity in sports science, with applications in injury risk analysis (15–19), sports performance analysis (16, 20), sports match prediction (21, 22), and match strategy development (23, 24). Machine learning, with its ability to learn from large datasets, enhance predictions, and formulate robust strategies (25), is anticipated to significantly enhance the objectivity of decision-making in sports science over the next decade (17–19).

Clearly understanding the impact of various factors and accurately predicting performance are crucial for developing effective training programs and optimizing athlete strategies, ultimately leading to competitive success (26, 27). Presently, machine learning techniques have seen preliminary application in the field of speed skating. For instance, Gao (28) utilized a genetic neural network algorithm for data training based on the results of women's speed skating competitions across the first 17 Winter Olympics. This approach enabled the prediction of competition outcomes for the 18th to 21st Winter Olympics, yielding high accuracy and practicality. Smyth and Willemsen (29) used case-based reasoning to study how environmental factors like altitude, track type and ice conditions affect speed skaters' performances, aiming to help athletes optimize their rhythm. Liu et al. (30) analyzed 16 seasons of ISU women's all-around speed skating data with machine learning algorithms, identifying the support vector machine as the best predictor of performance, especially in predicting advancement to finals based on 3,000 m event results.

This research leverages authoritative international competition data provided by the International Skating Union (ISU), applying the Back Propagation (BP) Neural Network algorithm (31, 32) to construct a comprehensive predictive model for speed skating performance. This technique, utilizing error backpropagation for the training of multilayer feedforward networks, is acclaimed for its exceptional predictive accuracy and robust generalization capabilities (33). By conducting an in-depth analysis of various influential factors, with a particular focus on ice surface temperature and competition frequency, our study introduces a quantitative approach to discern their impacts. This elucidates the underlying patterns that are instrumental in optimizing athletes' performances and in devising innovative training strategies. The fusion of machine learning and data analysis not only propels the evolution of sports science but also fortifies objective decision-making within the realm of speed skating.

## 2 External factors influencing speed skaters' performance

### 2.1 General review

When analyzing the athletic performance of elite speed skaters, it is crucial to recognize that it is regulated by a combination of numerous influencing factors, mainly categorized into two: subjective and objective. The primary subjective factor encompasses the skater's technical level, including skating skills, movement coordination, and other personal abilities. The second factor is psychological quality, involving handling competitive pressure, maintaining self-confidence, and controlling attention. Another key factor is competition strategy, encompassing the development of a tactical plan and strategies such as when to accelerate or maintain a constant speed. Additionally, daily training and self-regulation, involving the ability to formulate and execute training plans, as well as self-monitoring and flexible adjustment of training programs, are equally crucial. Lastly, the importance that athletes attribute to competition also influences their self-performance.

The influence of subjective factors is intricate. Elite athletes possess a relatively stable level of skill and tactics, resulting in limited subjective influence. Therefore, the impact of objective factors cannot be overlooked. These factors primarily stem from the external environment and conditions, as elaborated on the International Skating Union's (ISU) esteemed website. They encompass elements like ice rink altitude, ice temperature, game time, ambient temperature of the rink, and details about the inner and outer lanes, among others. External influences differ based on individual characteristics, duration, and intensity of the activity and necessitate thorough analysis.

Higher ice rink altitude, associated with lower air pressure and oxygen concentrations, as observed by Malashenkova and Romova (34), can potentially hinder a skater's endurance and aerobic capacity, leading to quicker fatigue and reduced performance at high intensity levels. Conversely, athletes acclimatized to higher altitudes undergo physiological adaptations, such as enhanced erythropoiesis and increased oxygen utilization, leading to improved performance. Additionally, the decrease in air density at higher altitudes lessens skaters' resistance to movement. An intriguing study by Noordhof et al. (2) demonstrated that a decrease in air pressure at higher altitudes can enhance the likelihood of winning medals in long track speed skating events by up to 10% owing to diminished air resistance.

Ice temperature and hardness are crucial factors. Higher ice temperatures reduce hardness, increasing friction and affecting skidding speed, while enhancing grip for high-speed cornering, starting, and stopping. Conversely, lower ice temperatures create a harder surface, diminishing agility and grip. The precise ice temperature is vital, and existing literature establishes a correlation between ice temperature and the coefficient of friction (10); Du et al. (35) have further contributed to theoretical understanding.

**Figure 1:**
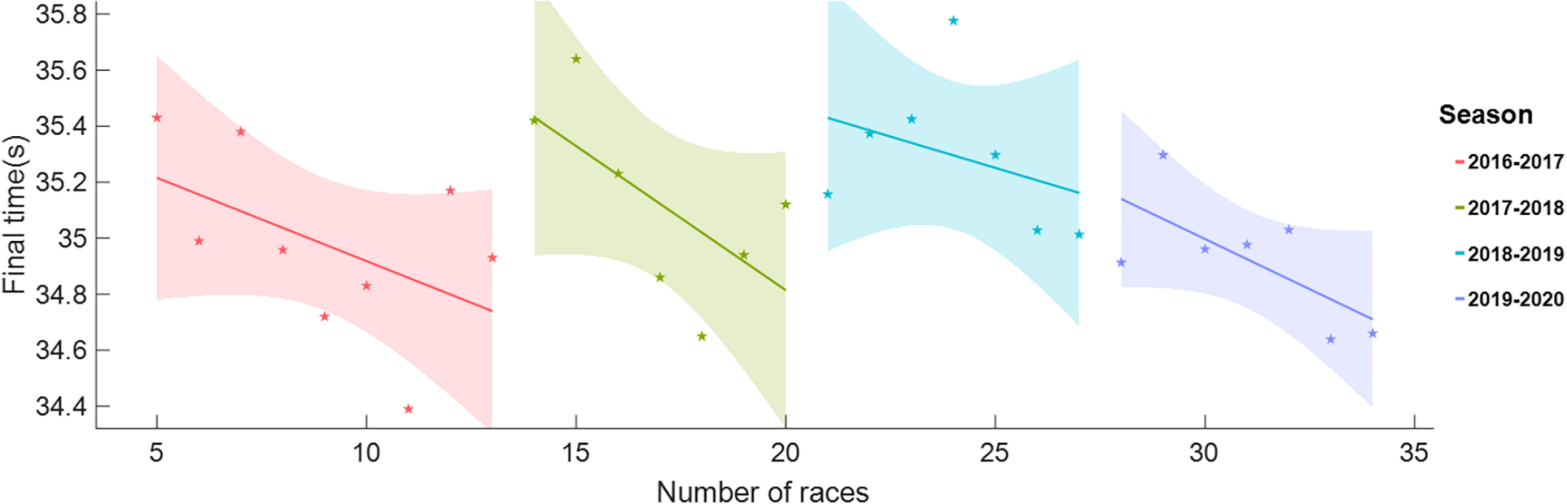
Variation of Tingyu Gao's race performance with the race number played in a season from 2016 to 2017 season to 2019–2020 season.

Race frequency, defined as the number of races a skater attended in a single season, exhibiting contrasting influences on skating performance. While it fosters skill development, experience, and performance enhancement, it concurrently imposes greater demands on skaters, necessitating heightened adaptability and skill execution, posing a risk of fatigue, and increasing the likelihood of injury. Striking a careful balance, meticulous race preparation, and adopting a comprehensive approach to enhance skater fitness are crucial to maximize the benefits and minimize the potential drawbacks of race frequency. Illustrated in Figure 1, the low-altitude race data of skater Gao Tingyu from 2016 to 2017 season through the 2019–2020 season, for instance, demonstrated improved performance with an increasing number of races in a single season. The shaded areas indicate 95% confidence intervals.

The evenness of the ice surface plays a pivotal role in the performance of speed skaters, and a well-maintained, flat surface is a prerequisite for optimal athletic performance. Irregularities such as bumps, cracks, or unevenness can significantly impede skating speed and pose safety risks. International ice rinks are renowned for their excellent ice quality. For instance, China's National Speed Skating Arena boasts an ice sheet flatness of ≤3 mm over an expansive 12,000-square-meter area, surpassing the strict ≤5 mm requirements set by the International Skating Union. Additionally, the positioning of skaters in the inside and outside lanes of the track profoundly influences their performance in various ways. The inside lane (lane 1) offers a shorter skating route than the outside lane (lane 2), providing an advantage for optimal performance. However, in group events, the outside lane is easier to overtake. Additionally, the psychological effects of track position must be considered. Analysis by Kamst et al. (36) demonstrated that the disadvantage of the inside lane at the end of the 500 m race had not been statistically significant since 2002. The controlled ambient temperature of a closed ice rink can also affect the performance of speed skaters. Lower temperatures typically help maintain a stiff ice surface, reducing friction but may lead to stiffness and cooling of the athlete's muscles, thereby affecting performance.

In conclusion, each of the factors we've examined profoundly influences the performance of speed skaters. In contrast to subjective factors, external objective factors can be precisely quantified and assessed, enabling the identification of crucial elements through scientific analysis. A comprehensive assessment of these factors is crucial for scientifically advancing the competitive level of speed skating.

### 2.2 Dominant factors based on kinematic model

Drawing on the energy balance model (37, 38), we can establish a kinematic model that describes the entire skating process of skaters, taking into account the influence of equivalent propulsive force, air drag, and friction, as shown in equations (1)–(3).

(1) $$\mathrm{ma}=\frac{P_{0}}{v}-F_{\mathrm{air}}-F_{f}$$

(2) $$F_{\mathrm{air}}=\frac{1}{2}\rho C_{d}Av^{2}$$

(3) $$F_{f}=\mu \mathrm{mg}$$

Here, *m* represents the skater's mass, *a* is acceleration, $P_{0}$ is the propulsion power, *v* is the relative velocity of air to the skater, $F_{\mathrm{air}}$ is air resistance, and $F_{f}$ is friction force. ρ is air density, $C_{d}$ is the drag coefficient, *A* is the skater's frontal area, and μ is the ice friction coefficient.

The kinematic model makes it evident that the primary resistances encountered in skating are air resistance $F_{\mathrm{air}}\;$ and friction resistance $F_{f}$. Air resistance is directly proportional to air density, and the ambient temperature at international high-level competition venues remains relatively stable and consistent, making the influence of the venue's altitude particularly pronounced (4, 39). The ice friction coefficient is predominantly influenced by the quality of the ice surface. In international high-level competitions, the ice surface is well-leveled, and the key factors affecting the friction coefficient are the temperature and hardness of the ice surface. Variations in ice temperature affect the contact area between the skater's blade and the ice surface (40) and the coefficient of friction (10), ultimately influencing frictional resistance. Propulsive power $P_{0}$ is influenced by subjective factors like the individual sporting ability. However, as previously mentioned, frequent races during the season can alter the skater's real-time condition, potentially leading to the accumulation of fatigue due to insufficient recovery time, thereby affecting energy output during races. Hence, our focus will be on the dominant external factors contributing to changes in these three parameters, namely: ice rink altitude, ice surface temperature, and race frequency.

## 3 Methods and verification

### 3.1 An analyse framework based on BP neural network model

#### 3.1.1 Neural network model structure

**Figure 2:**
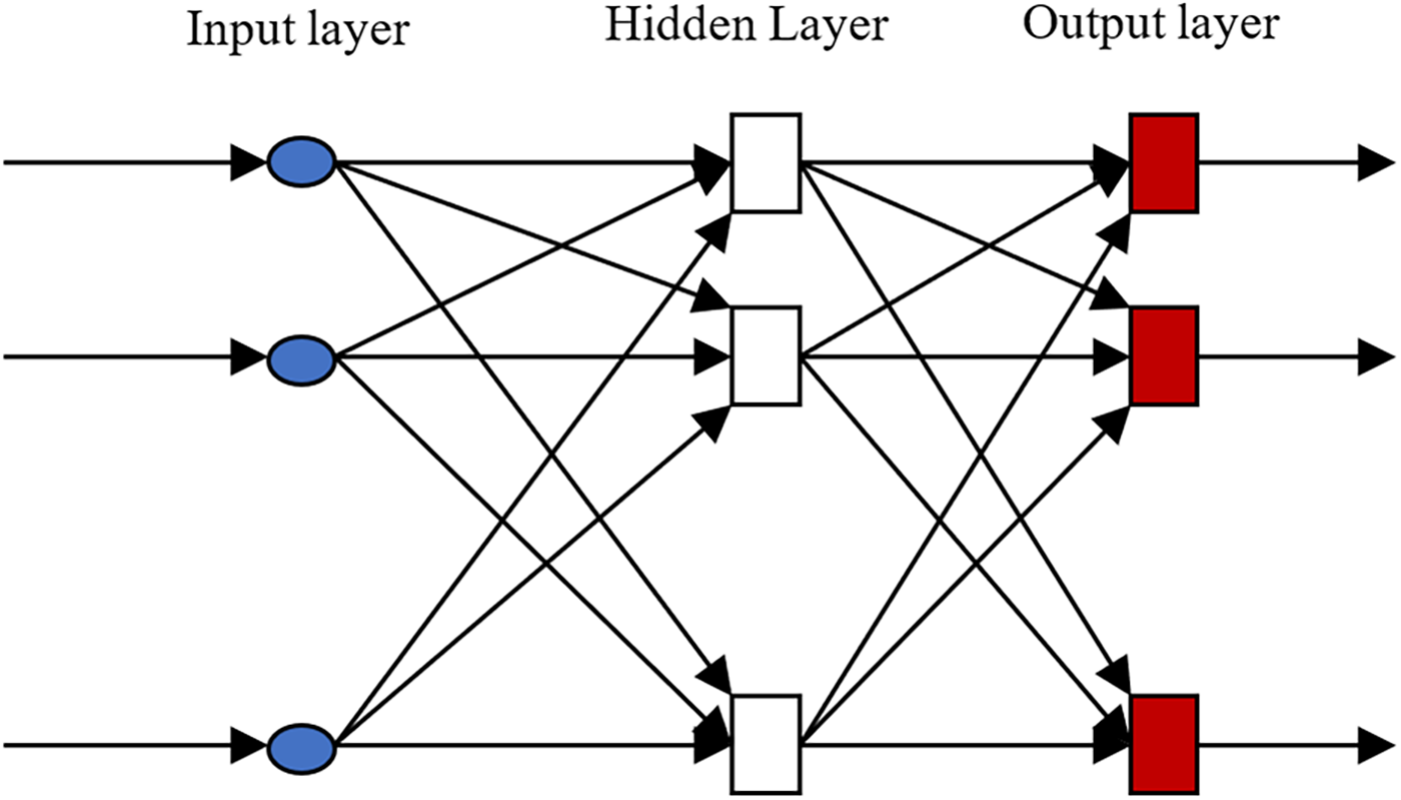
Structure of BP neural network.

**Figure 3:**
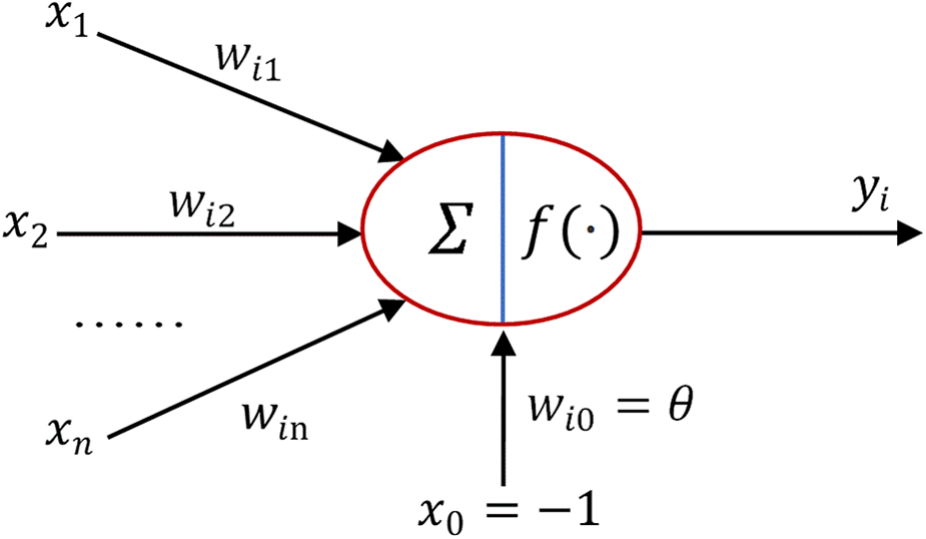
Neuronal structure.

In Figure 2, the back propagation (BP) neural network consists of three main components: the input layer, hidden layer, and output layer. The neural architecture within the BP neural network is illustrated in Figure 3. $x_{1},x_{2},\cdots ,x_{n}$ represent input signals received from other neurons; $w_{ij}$ denotes the connection weight from neuron *j* to neuron *i*; θ represents a threshold (Threshold) or bias (Bias); Σ denotes summation; and *f* represents the activation function. The designed neural network model employs the mean squared error as the loss function, with the Sigmoid function chosen as the activation function.

#### 3.1.2 Dataset and preprocessing

We obtained data from the International Skating Union (ISU) official website on 8 elite speed skaters representing the Chinese National Speed Skating Team in the Speed Skating World Cup and Winter Olympic Games for at least the last three years (three male, five female). The competition period spans from 2013 to 2022, with each athlete having a varying number of competition records, ranging from 13 to 105 ensuring a comprehensive representation of their competitive history. Removing data with anomalies helps ensure the reliability and accuracy of the study. We excluded data where skaters had a clear failure to perform during a race due to the effects of injury or illness, or where the result of a race was cancelled by the officials due to foul play.

Data sets for each skater, arranged chronologically, provide a comprehensive view of each skater's performance throughout their careers. This range allows for an exploration of performance trends and potential correlations with influencing factors. Overall, utilizing a sizable and reliable dataset from reputable sources is crucial for conducting a thorough analysis and drawing meaningful conclusions about the performance of speed skating skaters.

It is noted that because of the presence of training years, there is an overall difference in the performance of skaters in different seasons. In this study, the data from all seasons will be fused by introducing the method of eliminating training years from “relative final time” expressed in equation (4), so that the results of data analysis can reflect the pattern of the influence of external factors throughout the skaters' sporting careers and improve the prediction accuracy of the BP neural network.

(4) $$t_{\mathrm{rel}}=\frac{t_{\mathrm{abs},i}}{\frac{1}{n}\Sigma_{1}^{n}t_{\mathrm{abs},i}}$$

In this context, $t_{\mathrm{abs},i}$ signifies the absolute final time achieved in the i-th race during a specific season, with *i* ranging from 1 to *n*, where *n* represents the total number of races within that season. Table 1 provides a summary of the 9 variables extracted from the public dataset utilized in this study.

**Table 1:** Variables and samples.

Variables		Samples	Units
$t_{\mathrm{abs}}$	Absolute final time	e.g., “34.32”	s
$t_{\mathrm{rel}}$	Relative final time	e.g., “1.0014”	
A	Ice rink altitude	e.g., “49”	m
T	Ice temperature	e.g., “ −7”	°C
R	Race frequency	e.g., “3” represents the third race in a season	
$T_{\mathrm{air}}$	Temperature	e.g., “15”	°C
H	Humidity	e.g., “35”	°C
D	Race date	e.g., “20220212”	
L	Inner/Outer lane	e.g., “1” or “2” represents the inner or outer lane	

The prerequisite for data normalization prior to training is imperative, given the substantial disparities in magnitudes observed among the variables present in the raw dataset. This entails rescaling all data to the standardized range of 0 to 1, a procedure delineated in equation (5).

(5) $$X=\frac{x-x_{\min}}{x_{\max}-x_{\min}}$$

The Mean Absolute Percentage Error (MAPE) is a vital metric for neural network model assessment, as illustrated in equation (6).


(6) $$\mathrm{MAPE}=\frac{1}{k}\sum_{t=1}^{k}\left|\frac{A_{t}-F_{t}}{A_{t}}\right|\times 100$$

Here, *k* represents the number of testing and training datasets, $A_{t}$ stands for the actual value, $F_{t}$ represents the predicted value through neural network. In this context, uppercase E and lowercase e represent MAPE for testing and training datasets, respectively. When evaluating the model, it's crucial to balance the model's fitting capability for a particular combination and its corresponding prediction accuracy.

In this study, we introduce the parameter *Δ*, as outlined in equation (7), and assess its significance across various scenarios. A lower *Δ* is indicative of superior performance. Utilizing this metric, we make informed decisions regarding the selection of input and output metrics for the neural network model, determining the optimal number of neuron nodes in the hidden layer, and identifying high-performing neural network models suitable for network aggregation.

(7) $$\begin{array}{c} \Delta =E+e \end{array}$$

The model underwent optimization through parameter adjustments and the exploration of various combinations of input and output parameters. The results show that when the input parameters are ice rink altitude, ice surface temperature and race frequency, and the output parameter is relative final time, the Δ obtained according to equation (7) is the smallest, which is exactly the same as the important influencing factors we obtained through theoretical analyses. Therefore, we elected to these indicators as input factors, while utilizing relative final time as the output factor to construct our neural network model. This strategic selection aims to enhance the model's robustness and applicability.

#### 3.1.3 Determining hyperparameters

The number of nodes in the hidden layer of the BP neural network model is determined by equation (8) (41), where *m* represents the hidden layer nodes, *n* stands for the input layer nodes, *l* is the output layer nodes, and α is a constant within the range of 1 to 10.

(8) $$m=\sqrt{n+l}+\alpha \;$$

In our study, we set n=3 and l=1. Hence, *m* varies between 3 and 12. To mitigate the influence of chance, 10 training sessions were carried out for each node count. The corresponding Δ values for different node counts in the hidden layer are as follows: 1.17%, 1.16%, 1.20%, 1.16%, 1.23%, 1.22%, 1.19%, 1.13%, 1.14%, and 1.25%, respectively. We determined that the optimal number of hidden layers for skater GAO Tingyu's dataset is 10.

#### 3.1.4 Constructing the prediction matrix

In order to obtain the influence patterns of the three dominant factors, a continuous prediction matrix needs to be built. Firstly, for a single data point, the predicted relative final time, $t_{\mathrm{rel}}$, is calculated using the neural network model with input variables ($A_{0}$, $T_{0}$, $R_{0}$). Subsequently, the predicted absolute final time, ${\hat{Y}}_{0}$, is obtained by multiplying $t_{\mathrm{rel}}$ by the total career average final time of the corresponding skater, $t_{\mathrm{ave}}$, as illustrated in equations (9) through (11).

(9) $$t_{rel}=f(A_{0},T_{0},R_{0})$$

(10) $$t_{\mathrm{ave}}=\frac{1}{a}\Sigma_{1}^{a}t_{\mathrm{abs},i}$$

(11) $${\hat{Y}}_{0}=t_{\mathrm{rel}}*t_{\mathrm{ave}}$$

In these equations, the function *f* represents the neural network model, responsible for the processes of normalization, prediction, and recovery of input variables $\left(A_{0},T_{0},R_{0}\right)$. Meanwhile, $t_{\mathrm{abs},i}$ signifies the absolute final time of the i-th race within a skater's race dataset, where *i* ranges from 1 to *a*, and *a* denotes the total number of races in the dataset pertaining to a specific skater.

This study assesses the overall impact of a variable on race performance, specifically under conditions of low altitude, average ice temperature, and mid-season settings. Consequently, $A_{\mathrm{con}}$ is fixed at 50, while $T_{\mathrm{con}}$ and $R_{\mathrm{con}}$ are established at the respective mean values extracted from the skater's dataset, as expressed in equations (12) and (13), respectively.

(12) $$T_{cons}=\frac{1}{a}\Sigma_{1}^{a}T_{j}$$

(13) $$R_{cons}=\frac{1}{a}\sum_{1}^{a}R_{j}$$

In the context of the variables under investigation, $A_{\min},A_{\max},T_{\min},T_{\max},R_{\min}$, and $R_{\max}$ denote the minimum and maximum values pertaining to altitude, ice temperature, and the number of races participated in a season, all of which constitute the independent variable matrix. Specifically, $A_{\min}$ is standardized to 0, while $A_{\max}$ is set at 1,500 to simulate realistic altitude variations on the ice rink. $T_{\min}$ and $T_{\max}$ are determined from the respective minimum and maximum values observed in the ice temperature variable within a particular skater's dataset. Moreover, $R_{\min}$ is established at 1, and $R_{\max}$ corresponds to the highest number of races played across all seasons. To ensure the creation of a continuous prediction curve, the matrix is composed of 500 columns, encompassing 500 uniformly selected data points that span the range between the minimum and maximum values, so a prediction matrix can be built from multiple prediction points.

We can define the altitude influence matrix, $M_{A}$, by incrementing δ for the front and rear columns of the altitude variable. When $M_{A}$ is input into the neural network model, it yields multiple results, $\hat{Y}$, thereby forming the prediction curve. The altitude's influence slope can be determined by calculating the slope of this curve. equations (14), (15), and (16) provide the specific expressions:

(14) $$M_{A}=\left(\begin{array}{ccc} R_{\mathrm{con}} & R_{\mathrm{con}} & R_{\mathrm{con}}\\ T_{\mathrm{con}} & T_{\mathrm{con}} & T_{\mathrm{con}}\\ A_{\min} & A_{\min}+\delta & A_{\min}+2\delta \end{array}\;\;\;\;\begin{array}{cc} \cdots & R_{\mathrm{con}}\\ \cdots & T_{\mathrm{con}}\\ \cdots & A_{\max} \end{array}\right)$$

(15) $$\hat{Y}=N(M_{A})*t_{\mathrm{ave}}$$

(16) $$k_{A}=K(\hat{Y})$$

$\hat{Y}$ represents the set of 500 predicted results, and the function *K* calculates the slope of the curve formed by $\hat{Y}.k_{A}$ represents the slope characterizing the influence of altitude on performance, computed from the predicted results of a single neural network model, with units expressed as seconds per kilometer (s/km). Similarly, $k_{T}$ and $k_{R}$ can be determined, with units denoted as seconds per degree Celsius $\left(s{/}^{\circ }C\right)$ and seconds per game (s/game), respectively.

#### 3.1.5 Neural network model aggregation

**Figure 4:**
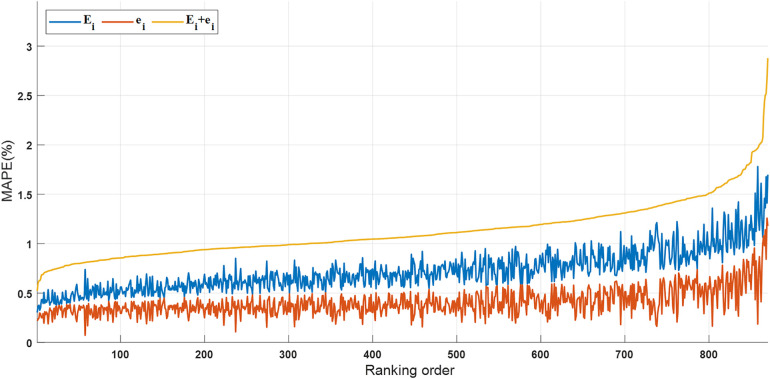
The curves of $E_{i},\;e_{i},\;\Delta_{i}$ of the network model sorted in ascending order of $\Delta_{i}$.

Neural network models are renowned for their nonlinear characteristics, rendering them susceptible to singularity and randomness errors, particularly when dealing with limited datasets. To mitigate this concern, this paper leverages ensemble learning and an aggregation model (42), as referenced in formula (7), which selects multiple high-performing neural network models and computes their average output after multiple calculations. The neural network model evaluation metrics for the *i*-th training iteration on the same dataset, including $\Delta_{i}$, $E_{i}$ and $e_{i}$, are delineated in the formula. For instance, taking skater Gao Tingyu's dataset as an illustration, with a hidden layer node count of 10 and training iterations ranging from i=1 to 1,000, the number of retained neural network models, following the removal of instances where $e_{i}>E_{i}$ (where $E_{i}$ generally exceeds $e_{i}$ in the *i*-th training), amounts to 870. The plot of neural network model evaluation metrics, sorted by $\Delta_{i}$ in ascending order, is depicted in Figure 4.

$K_{A,b}$, $K_{T,b}$ and $K_{R,b}$ are defined as the average slopes representing the influence of three factors on final time. These average slopes are computed based on predicted slopes obtained from multiple neural network models. The calculation method for $K_{A,b}$ is outlined in equation (17).

(17) $$K_{A,b}=\frac{1}{b}\Sigma_{1}^{b}k_{A,(j)}$$

Here, the subscript *j* of $k_{A,(j)}$ denotes the altitude influence slope predicted by the neural network model associated with evaluation indicator $\Delta_{i}$ located at the j-th position in ascending order, where *j* ranges from 1 to *b*, and *b* represents the total number of selected neural network models. The calculation methods for $K_{T,b}$ and $K_{R,b}$ are identical to the equation (16) presented earlier. Parameter independence analysis reveals that the observed trend remains stable when the number of neural network models reaches 100. Therefore, this paper opts for b=200.

#### 3.1.6 Summary of the overall algorithm framework

**Figure 5:**
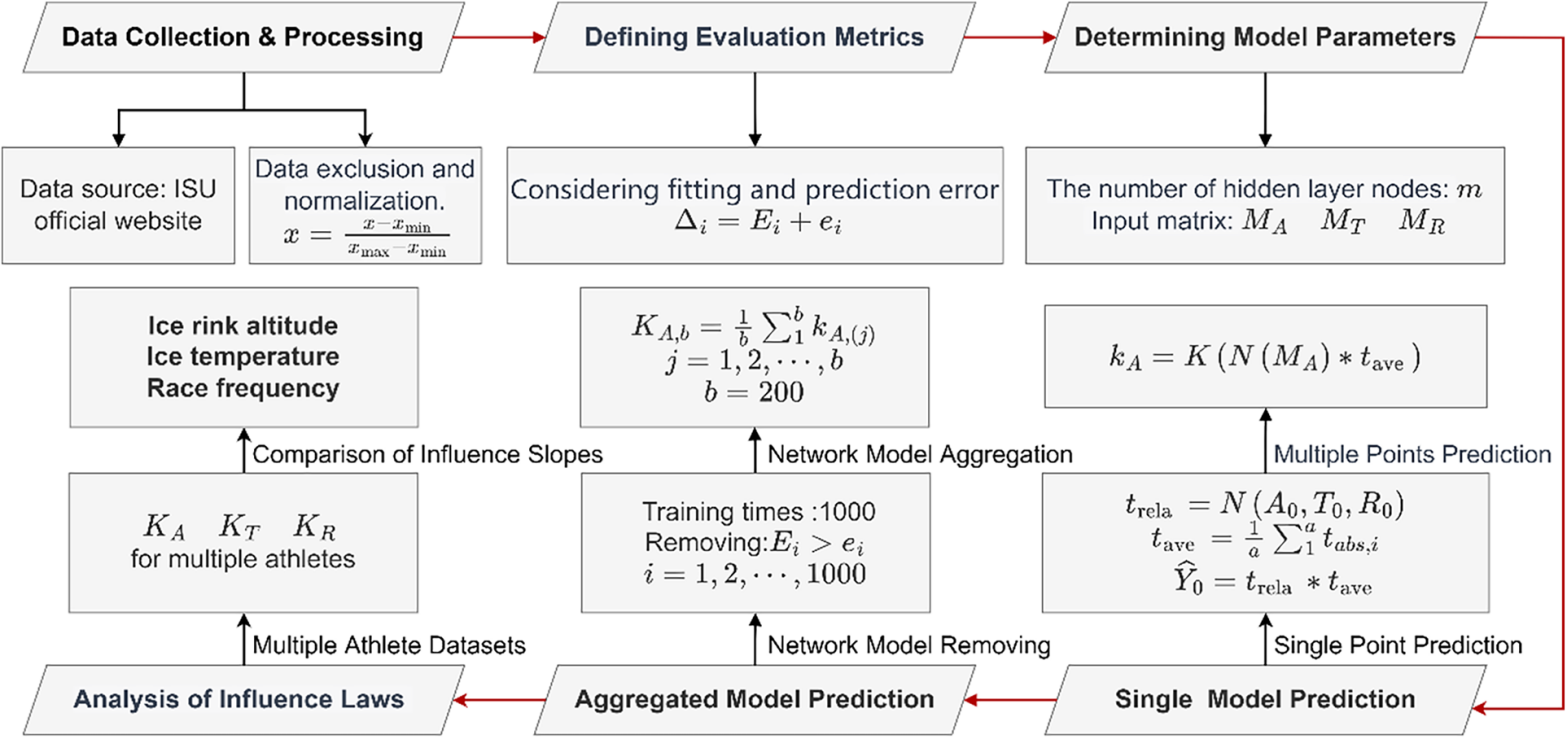
Flow chart of model establishment.

In order to compare and analyze variations in the impact of three factors on different skaters, we identify appropriate node numbers for each skater's dataset pertaining to various race distances. Subsequently, we select high-performing neural network models for predictions to derive $K_{A}$, $K_{T}$, and $K_{R}$. The influence of parameters on the overall algorithm framework is visualized in Figure 5.

### 3.2 Algorithm verification

**Figure 6:**
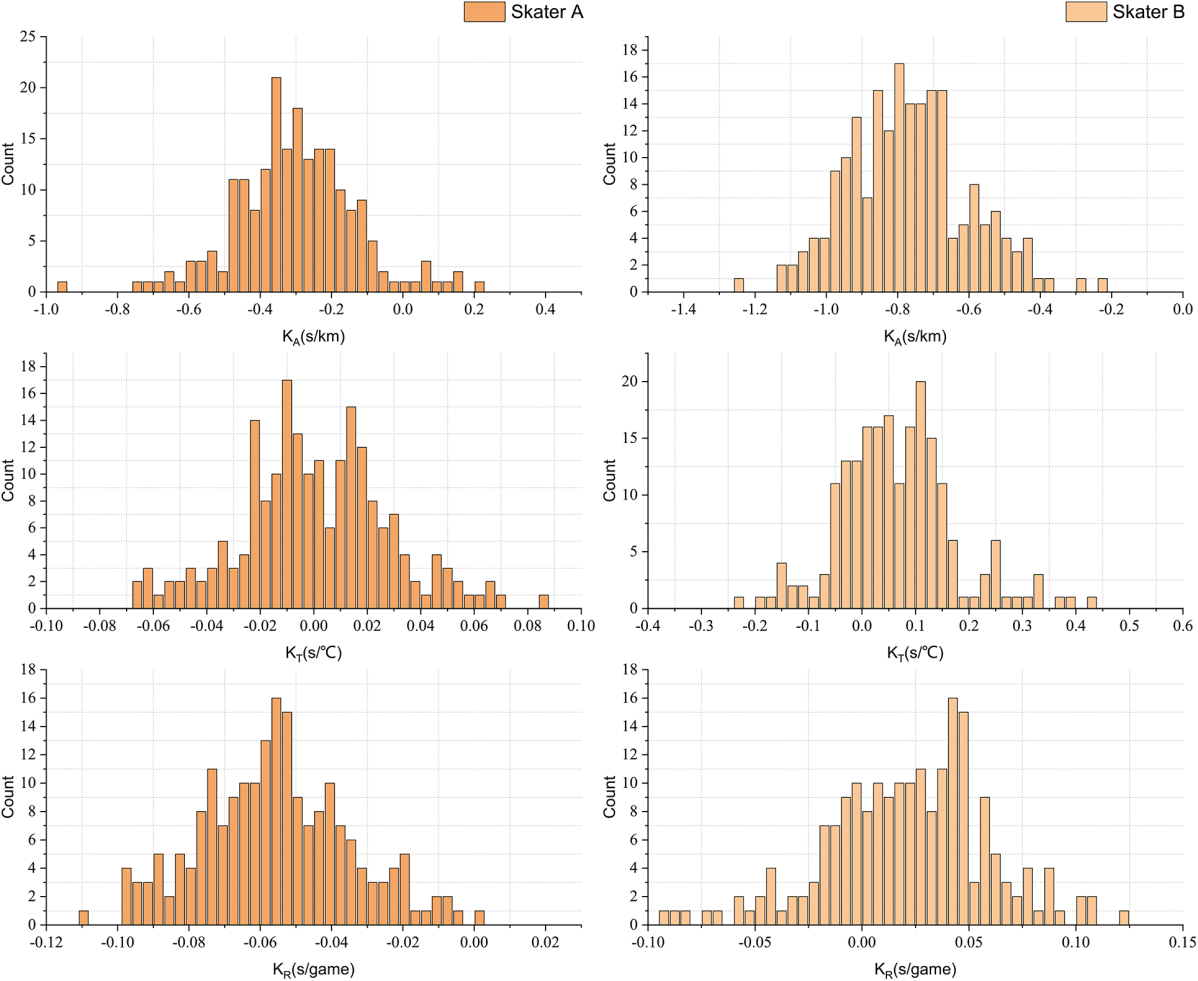
The slope frequency distribution histogram of the final time of player A and B influenced by three influencing factors.

Building upon the model described earlier, Figure 6 presents a histogram illustrating the frequency distribution of slopes pertaining to altitude, ice temperature, and race frequency, all of which influence the 500 m race final time for skaters A and B. Notably, the majority of altitude influence slopes exhibit negative values, suggesting that higher altitudes generally correlate with improved race performance. For skater A, the slope is predominantly concentrated between −0.75 s/km and −0.25 s/km, while for skater B, it falls mainly between −1.15 s/km and −0.3 s/km. These findings align with both theoretical analysis and practical experience outcomes. Conversely, the influence of the other two factors, namely ice temperature and race frequency, appears relatively minor, with their impact being less pronounced compared to altitude. This observation is consistent with theoretical predictions. These results substantiate the efficacy of the algorithm.

Moreover, the frequencies of the three slopes for both skaters displayed a trend resembling a normal distribution, with a concentration around the mean values of each slope.

Table 2 presents the mean slope values for the three influencing factors, offering valuable insights into the distinctive performance characteristics of skaters A and B. It is evident that rink altitude and ice temperature exert a substantial influence on skater B, whereas race frequency plays a significant role in skater A's performance.

**Table 2:** The average slopes of the race performance of player A and B influenced by three factors.

Skaters	Gender	K		
		$K_{A}$	$\;K_{T}$	$K_{R}$
A	Male	−0.3011	0.0004	−0.0557
B	Female	−0.7685	0.0654	0.0217

As illustrated in Figure 6, skater A's performance demonstrates a gradual improvement with an increasing number of races played within a season, aligning with the observations made in this study. In contrast, skater B's performance tends to exhibit better results in the early stages of the season, as indicated by the positive slope value.

### 3.3 Assessment of dominant external factors

In order to mitigate the impact of the original data dimensions, influence values are derived from the dimensionless slopes and $K_{R}$ to quantify the degree of influence. The altitude influence values can be determined using equation (18), as shown below.

(18) $$N_{A,i}=\frac{cK_{A,i}}{\Sigma_{1}^{c}|K_{A,i}|}$$

In this scenario, *c* represents the number of samples, which is 15 while $K_{A,i}$ signifies the raw calculated slope value influenced by the altitude for each sample. Similarly, $N_{T,i}$ and $N_{R,i}$ are obtained as the influence values of ice temperature and race frequency.

## 4 Results and discussion

The results of these calculations are presented in Table 3. The designations “Skater A-H” represent the 15 datasets derived from the participation of the 8 skaters in our study, with an asterisk denoting a female skater. To delve deeper into valuable insights, we computed dimensionless influence values utilizing the findings derived from an extensive dataset featuring 8 diverse skaterss participating in varying race distances, totaling 15 races. Subsequently, we conducted succinct influence ratings and analyses rooted in these values, aiming to uncover the distinctive characteristics of external influences on the performance.

**Table 3:** Influence values of the three influencing factors.

Sample number	Skaters	Race distance	N		
			$N_{A}$	$N_{T}$	$\;N_{R}$
1	A	500 m	−13	0	−10
2	B*	500 m	−34	44	4
3	C	500 m	−16	19	−9
4	D*	500 m	−18	−6	0
5	E	1,000 m	−89	15	−4
6	D*	1,000 m	−78	−16	−2
7	F*	1,000 m	−81	36	−109
8	E	1,500 m	−141	80	−13
9	G*	1,500 m	−105	−28	−196
10	D*	1,500 m	−128	63	−31
11	F*	1,500 m	−138	29	−99
12	H*	1,500 m	−78	−121	−122
13	F*	3,000 m	−98	134	−221
14	G*	3,000 m	−214	−510	−187
15	H*	3,000 m	−270	−399	−494

### 4.1 Ice rink altitude

**Figure 7:**
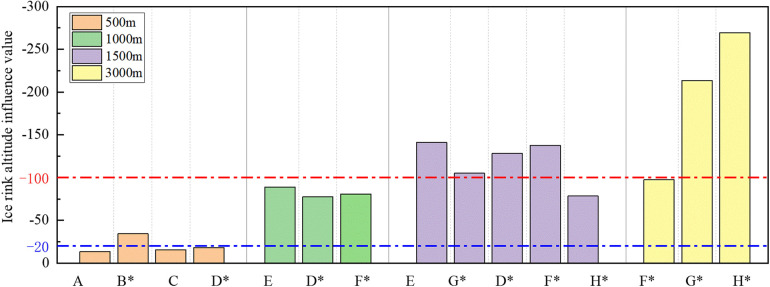
Bar chart of ice rink altitude influence values and the boundary line of influence rating.

Utilizing the absolute influence values from Table 3, we assessed the degree of the influence of the three dominant factors on race performance. Categorizing influence ratings as negligible, perceptible, and important based on the ranking of absolute influence values in the top 33%, middle 33%, and bottom 33%, with corresponding boundary values of 20 and 100. Figure 7 visually presents the influence values of ice rink altitude, along with the influence ratings and their boundary lines. The rink altitude ranges from 0 to 1,425 m. Table 4 was generated from Figure 7, providing statistical results of altitude influence ratings for different playing distances.

**Table 4:** Statistical table of influence ratings of ice rink altitude.

Factors	Ice rink altitude			
Distance	0.5 km	1 km	1.5 km	3 km
Negligible	3	0	0	0
Perceptible	1	3	1	1
Important	0	0	4	2

Figure 7 illustrates that higher rink altitudes correspond to an enhancement in race performance. This observation aligns with previous studies (2, 3, 4). For instance, Muehlbauer et al. (5) demonstrated that at high altitudes, skaters exhibit shorter time per split lap (*p* < 0.001), resulting in a shorter total time. The reduced oxygen supply at high altitude and its impact on energy output have been crucial discussion points. While high altitude leads to decreased oxygen supply, the findings suggest that, within the altitude range of speed skating rinks (0–1,425 m), high altitude does not negatively affect performance due to reduced oxygen supply. van Ingen Schenau et al. (43) also concluded that at altitudes of 2,000 m or less, a reduction in oxygen consumption is unlikely to significantly impact speed skating. Therefore, the dominant factor influencing the performance of all skaters is the reduction in aerodynamic resistance due to high altitude.

Table 5 indicates that ice rink altitude can have a perceptible or even important influence on performance in races of 1,000 m and above. In the 1,000 m races, altitude's influence is consistently perceptible; in the 1,500 m races and beyond, altitude is more likely to exert an important influence. Conversely, altitude influence is predominantly minor in the 500 m races, underscoring the growing importance of altitude with increasing race distance. A prior study by Muehlbauer et al. (5) revealed significant time differences in race times at high and low altitudes for women's 3,000 m and men's 5,000 m races, amounting to 11.75 s (*p* < 0.001) and 24.44 s (*p* < 0.015), respectively. Another study (2) demonstrated that altitude's influence on junior skaters' performance in short (500 m and 1,000 m) and long (1,500 m and 3,000 m) races was 2.5% and 3.2%, respectively. Additionally, the performance prediction model proposed by van Ingen Schenau et al. (4) indicated that higher altitude resulted in a more pronounced time advantage with increasing race distance, validating our findings. Clearly, since shorter race distances consume less time, athletes experience a shorter duration in a low air density environment. Therefore, in the shortest distance race of 500 m, cases where the influence of altitude was negligible were predominant. As the distance increases and the duration of the race extends, the time gain from altitude amplifies.

**Table 5:** Statistical table of influence ratings of ice temperature.

Factors	Ice temperature			
Distance	0.5 km	1 km	1.5 km	3 km
Negligible	3	2	0	0
Perceptible	1	1	4	0
Important	0	0	1	3

Researchers have observed that a 1,000-m increase in altitude boosted the average performance of junior skaters by 2.8%, with high-level athletes experiencing a slightly lower increase of 2.1% (2). This discrepancy is likely attributed to their differing levels of proficiency: junior skaters, with fewer races and an underdeveloped technical and tactical system, contrast with elite skaters who benefit from better race preparation, greater experience, and more consistent motivation derived from top athletes, making them less susceptible to external factors. Additionally, there is inherently less internal variability among senior athletes. For instance, among junior skaters, female athletes exhibit higher internal variability than males, whereas among elite athletes, the difference between males and females is negligible.

In conclusion, elevating altitude will positively affect the performance of elite athletes, with the influence becoming more pronounced as the race distance increases.

### 4.2 Ice temperature

**Figure 8:**
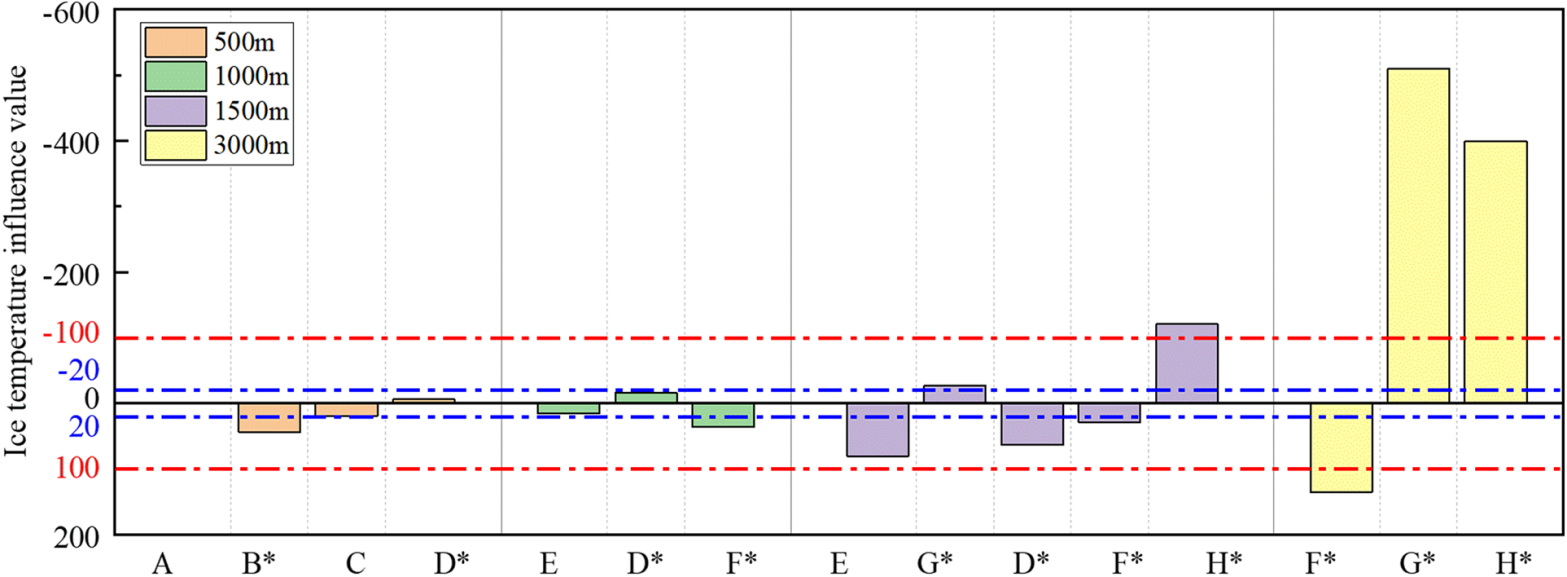
Bar chart of ice temperature influence values and the boundary line of influence rating.

Figure 8 illustrates the values and ratings indicating the influence of ice temperature, while Table 5 presents the influence ratings of the ice temperature across various race distances.

Figure 8 suggests that the influence of ice temperature is more individualized, with some skaters displaying better performance at lower ice temperatures, while others excel at higher ice temperatures. Despite limited studies on the influence of ice temperature on skating performance, the results indicate an uncertain relationship between ice temperature and race performance, likely varying among skaters with different skill profiles. As ice temperature decreases, the ice surface becomes harder, potentially reducing frictional resistance during skating (10). This change may benefit skaters focusing on longer skating times, applying higher pedal forces, and employing lower pedal stroke frequencies. Conversely, relatively high ice temperatures may favor skaters who adopt a higher frequency of pedal strokes and lower pedal force.

As per Table 5, the influence of ice temperature becomes perceptible in the 1,500 m race and importantly affects the performance time in the 3,000 m race.The influence is mainly minor in the 500 m and 1,000 m races, at 75% and 67%, respectively. In line with our own findings, simulations by de Koning and Schenau (44) indicated that the influence of ice temperature becomes more pronounced with increasing race distance. The amplification of this impact is attributed to the prolonged duration of its influence on race time, akin to that of ice rink altitude.

It is noteworthy that there is considerable consistency in the influence of ice temperature on most all-around skaters participating in races at various distances. For instance, skaters E and F consistently performed better at higher ice temperatures in the 1,000 m, 1,500 m, and 3,000 m races, while skaters G and H consistently performed better at lower ice temperatures in the 1,500 m and 3,000 m races. However, the influence of ice temperature on different distances may exhibit opposite trends in the same skater. For example, skater D performed better in the 1,500 m race when the temperature was lower and the ice was harder, whereas the opposite was true in the 500 m and 1,000 m races. Specifically, in a 500 m race one season, Skater D clocked 38.49 s at −4.8°C ice temperature compared to 39.21 s at −7.3°C, while in a 1,500 m race, she achieved a time of 117.081 s at −8°C ice temperature compared to 121.087 s at −6°C. This observation underscores the possibility that skaters tend to adapt different technical attributes to their strengths at different race distances.

**Figure 9:**
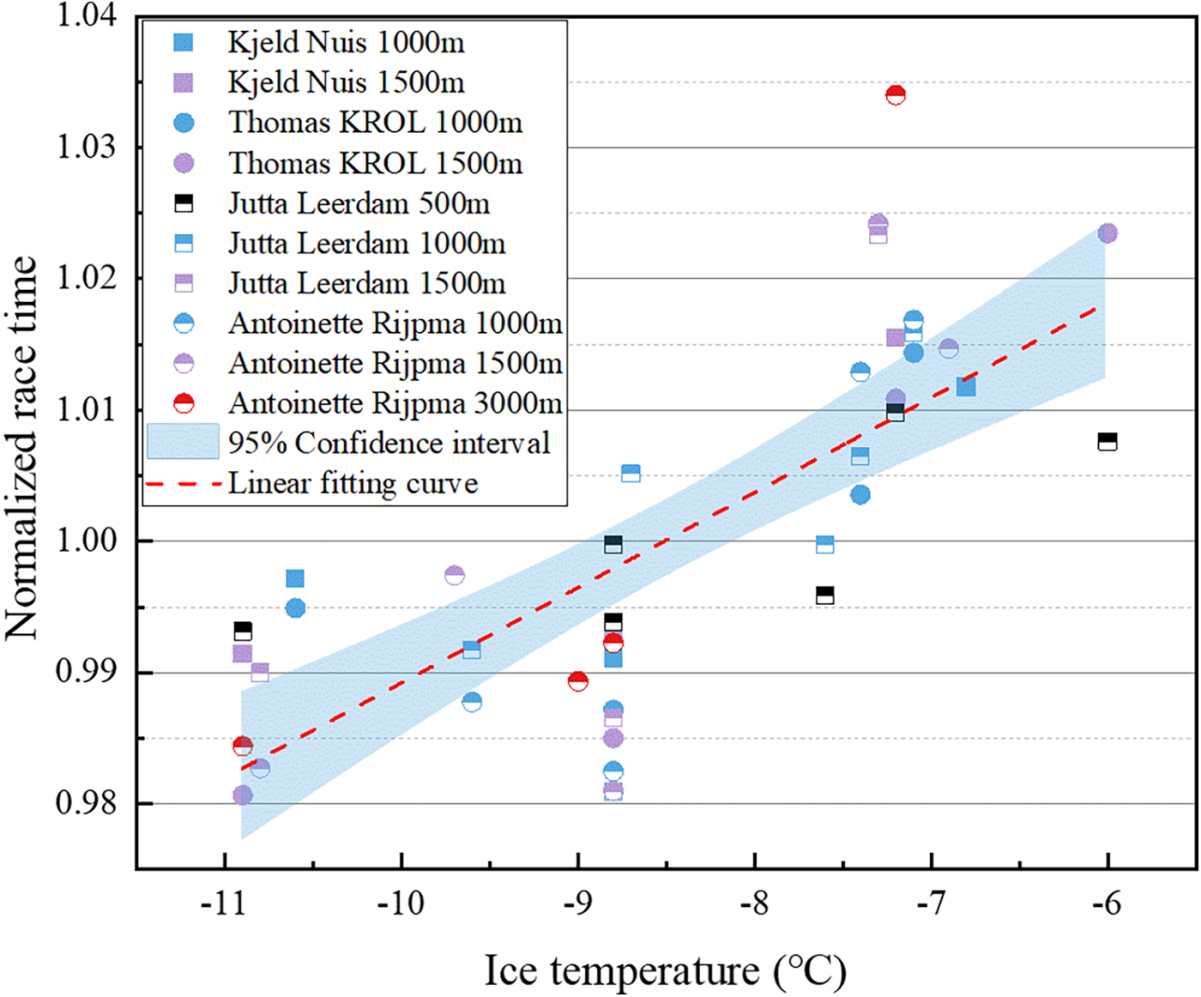
Relationship between normalized race time and ice surface temperature for the 4 Dutch all-around speed skaters.

To further validate the reliability of the results, we obtained actual game times and ice temperatures from the official website of the National Skating Union for Dutch high-level all-around male players Kjeld Nuis, Thomas KROL, and female players Jutta Leerdam, Antoinette Rijpma-de Jong, during the 2022–2023 season at low altitude rinks. To eliminate the impact of different race distances, we normalized the race times by dividing them by the average of the race times within the corresponding race distances. Figure 9 illustrates the relationship between standardized race time and ice temperature for the 4 Dutch all-around speed skaters, along with linear fitting curves. The figure indicates a significant positive correlation between performance time and ice temperature for the Dutch high-level athletes, consistently performing better at lower ice temperatures across all distances competed. Importantly, we did not observe a change in the correlation between performance and ice temperature for the same skater across different race distances, suggesting that these all-around high-level athletes exhibit relative stability in terms of their competition skill characteristics.

To sum up, the trend in the influence of ice s temperature is highly correlated with the technical characteristics of skaters, emphasizing the importance of considering individual technical and tactical attributes when assessing their influences. For instance, optimizing performance could involve engaging skaters with “ Prefer low ice temperatures” skill profiles in more low ice temperature races. Alternatively, in high ice temperatures, the skater should increase pedal frequency appropriately and minimize skating time on the ice. Conversely, in low ice temperatures, the opposite holds true, particularly in competitions exceeding 1,500 m. We recommend making technical adjustments tailored to the skater's performance on a given ice surface to fully capitalize on the rink conditions.

### 4.3 Race frequency

**Figure 10:**
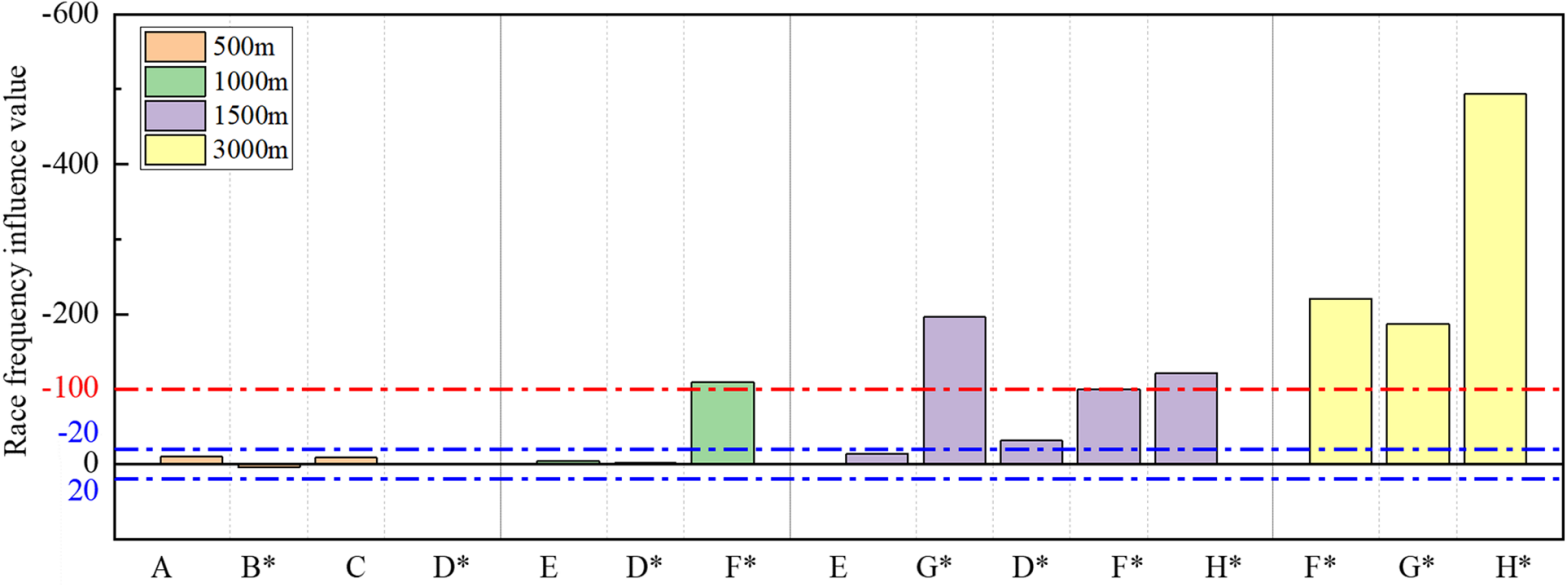
Bar chart of race frequency influence values and the boundary line of influence rating.

Figure 10 displays the influence values and influence ratings of race frequency, while Table 6 presents the influence rating results of race frequency within different distances.

**Table 6:** Statistical table of influence ratings of race frequency.

Factors	Race frequency			
Distance	0.5 km	1 km	1.5 km	3 km
Negligible	4	2	1	0
Perceptible	0	0	2	0
Important	0	1	2	3

A crucial observation from Figure 10 is that performance in most races improves with the increasing race frequency. This aligns with previous research advocating high pre-competition training loads, emphasizing the potential benefits of peaking before competition (12). Moreover, finals, semi-finals, and quarterfinals generally exhibit faster finishing times than the preliminary stages of the competition. The influence of race frequency on finishing time gradually diminishes as the race progresses (1). High-level skaters progressively enhance their competitive level, adapting to the race environment and pressure through the accumulation of races. The growing significance of races further enhances skaters' concentration. It is noteworthy that high-level skaters embrace a more scientific approach and rely on robust team safeguards during race preparation, enabling them to manage fatigue effectively and promote rapid body recovery. These efforts ultimately contribute to significant improvements in their athletic performance.

According to Table 6, the influence of race frequency on the performance of the 500 m race is negligible, while it is all important on the 3,000 m race, and its influence on other distance races is more variable. The total time of the 500 m race is the shortest. With the increasing race frequency, the time improvement in the 500 m race will inevitably be smaller compared to the 3,000 m race, which has a longer total time and more opportunities to demonstrate progress in technical movements. Additionally, there is a notable disparity in the magnitude of the influence of race frequency on different skaters within the same distance. In the 1,000 m and 1,500 m races, the influence of race frequency may range from important to minor. As previously mentioned, the varying technical progress and fatigue management levels among different skaters as race frequency increases result in diverse sensitivity to the influence. Consequently, the progress levels of each skater are also inconsistent, with considerable individual differences.

**Figure 11:**
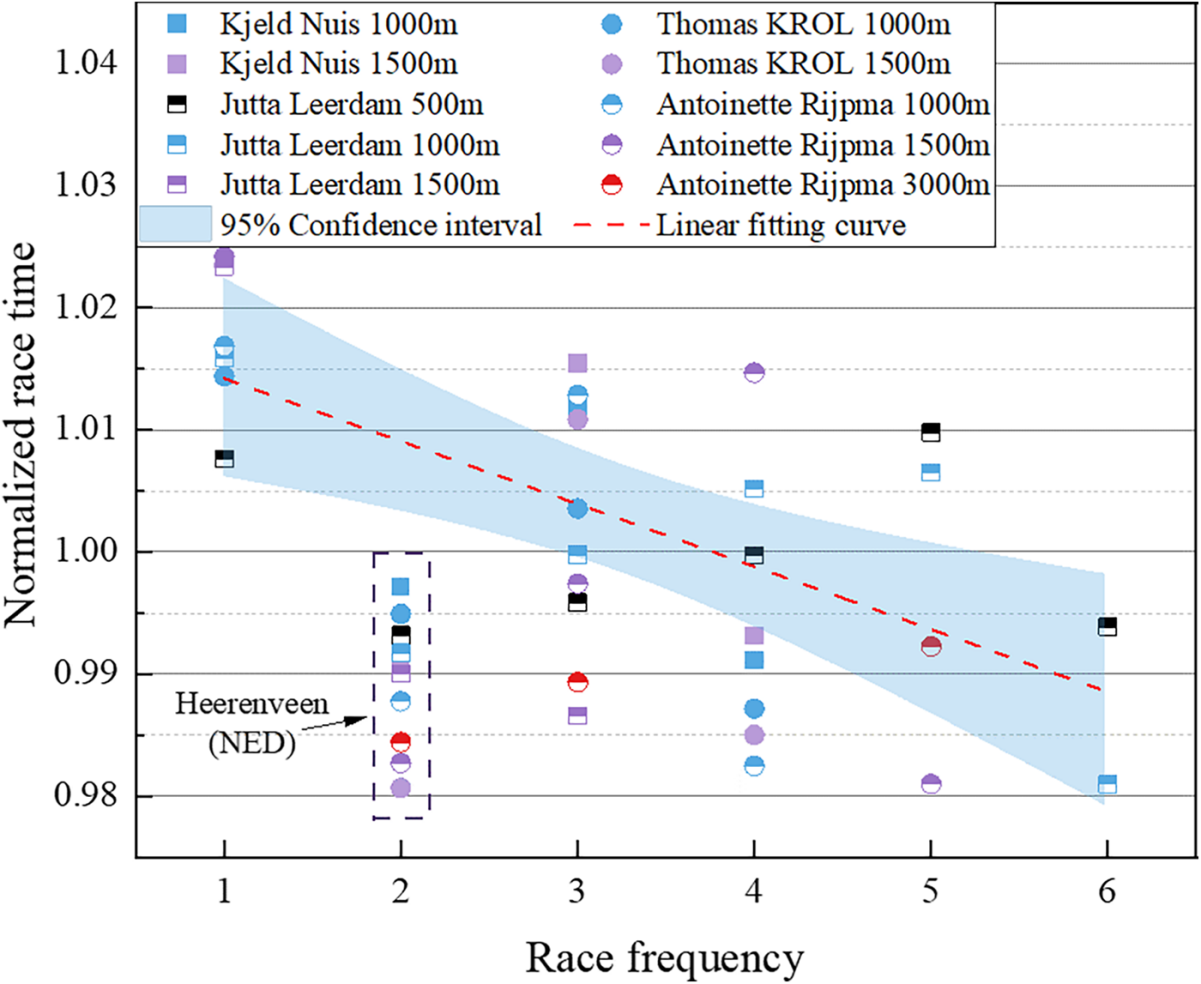
Relationship between normalized race time and race frequency for the 4 Dutch all-around speed skaters.

Similarly, we explored the relationship between the race results of the four mentioned Dutch skaters and the race frequency in the season. The data points were linearly fitted, as illustrated in Figure 11. The fitting curve indicates that as the race frequency increases, the skater's performance significantly improves, aligning with our analysis results. It is noteworthy that the dotted box in the Figure 11 corresponds to the second stop of the 2022–2023 season in Heerenveen, the Netherlands, for the World Cup, where the average ice surface temperature was as low as −10.56°C, 34% lower than the average ice temperature of −7.88°C in other games. The low ice temperature conditions may have contributed to their results being better than the average performance of the season, so the data is relatively smaller and was not considered in the fitting.

In summary, the findings suggest that race frequency has an overall positive influence on athlete performance, potentially through multifaceted mechanisms, especially in the 3,000 m races. However, significant individual differences exist, emphasizing the role of performance decline in some skaters and underscoring the importance of personalized monitoring. Therefore, increasing race frequency could be a viable strategy to enhance performance, provided that skaters' fitness and recovery levels are carefully monitored.

### 4.4 Comprehensive analysis

Figures 7–11 illustrate the unique influence characteristics of the three dominant external factors, effectively highlighting variations in the sensitivity of different skaters to external influences. Further comparative analysis allows a deeper exploration of these specific influence characteristics.

**Figure 12:**
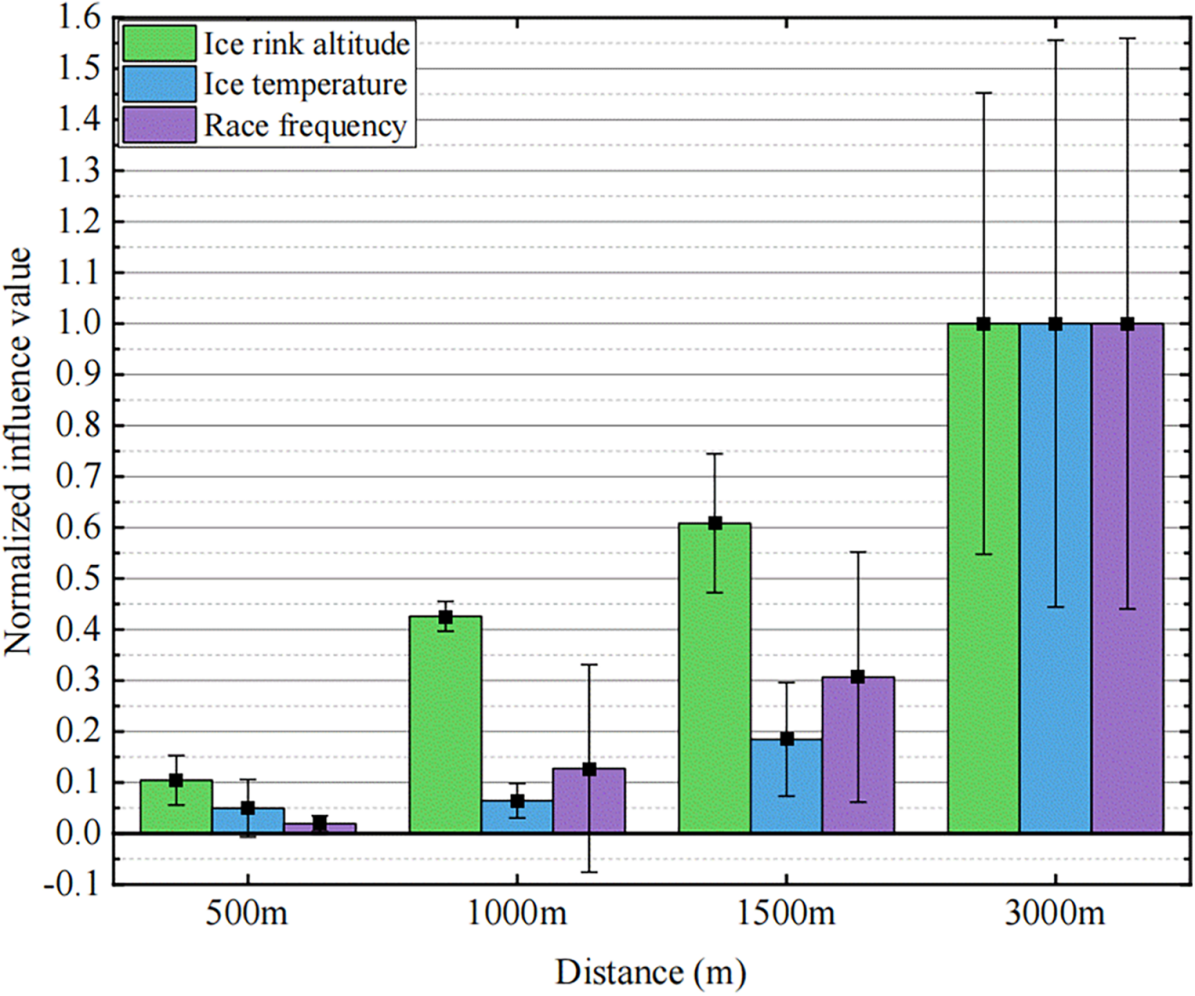
Normalized mean influence and the influence difference of ice rink altitude, ice surface temperature, and race frequency on skaters.

Utilizing the mean and standard deviation of the absolute influence values within a specific distance can characterize the overall average influence of external factors on skaters and the degree of difference in influence. Figure 12 illustrates the relationship average influence of three dominant external factors and the influence difference on skaters.

In this context, the average influence of each influencing factor on the 3,000 m race serves as a benchmark which is 1.The average influence values and the standard deviations of the influence values for other race distances are normalized. This approach enables a clear expression of the characteristics of the three influencing factors on different race distances.

The observation reveals that as the race distance increases, the influence of the three key external factors significantly intensifies. When the race distance reaches 1,000 m, we cannot overlook the advantageous influence of altitude. The distinction lies in the fact that, in comparison to other distances, the influences of ice temperature and race frequency are crucial for the 3,000 m race. Consequently, with increasing race distances, skaters must prioritize the influence of external factors and leverage them to enhance performance, particularly in races of 3,000 m and longer.

Combining Table 3 and Figure 12 reveals the distinctive influencing characteristics of the three factors. Firstly, regarding influencing trends, the consistency of ice rink altitude is the strongest, with all skaters performing better at high altitudes; an increase in race frequency also generally has a beneficial influence on performance; however, the influence trend of ice surface temperature varies from person to person. Secondly, considering the difference in the degree of the influence, the influence of altitude on different skaters within the same distance is the smallest, suggesting that the altitude's influence mechanism is straightforward, yielding clear and easily observable results, and is less susceptible to other factors. In contrast, race frequency and ice surface temperature exhibit significant individual differences due to their complex influencing mechanisms.

### 4.5 Limitations

The study recognizes that limitations in sample size and data precision may influence the accuracy of the analysis, and our conclusions might be influenced by the characteristics of the study sample and the selected group of skaters. However, with the accumulation of more high-precision data in the future, the analysis is poised to become more reliable and insightful. Consequently, further research should aim to include a larger number of athletes at different levels to validate this trend.

## 5 Conclusion

The speed skating performance of elite athletes is intricately influenced by the complex interplay of multiple factors. Traditional two-dimensional scatter plots only depict the overall influence trend, lacking the ability to exclude the influence of other potential factors on the results. Hence, there is an urgent need for an efficient algorithm to dissect this intricate relationship. This study constructs a quantitative evaluation model for each skater's dataset, based on a large number of high-performance neural network models, which are aggregated to dig deeper into the skaters' race datas and the potential connections between race performance and dominant external influencing factors. We obtained the influence law of game performance by external factors under the condition of maximizing the exclusion of the influence of other factors, which could not be reached by the traditional analysis method using a single neural network model (33). It unveils the influencing characteristics of three factors and the varying degrees of influence on different skaters. Finally, we validated the effectiveness of the algorithm proposed in this study through comparisons with existing literature and actual data, emphasizing its significant advantages in terms of accuracy over traditional analysis methods. The results highlighted several major factors influencing skating performance:

1. Ice rink altitude: Skaters experience improved performance with higher ice rink altitudes, especially in races of 1,000 m and above. The variation in the influence size among different skaters at the same race distance is not conspicuous.
2. Ice temperature: Ice temperature can either enhance or impair performance and varies in its influences based on skaters' technical characteristics. It has a perceptible or important influence on races of 1,500 meters and longer, while its influence on the shorter 500 m and 1,000 m races is generally minor.
3. Race frequency: Increased race frequency generally enhances skaters' performance. The influence on the 500 m race is negligible, while it is important for the 3,000 m race. The influence on the 1,000 m and 1,500 m races varies among individuals.

In summary, this study aims to identify and explore dominant external factors influencing speed skating performance, particularly those easily observed in publicly available datasets. The findings indicate that rink altitude, ice surface temperature, and race frequency significantly influence skaters' performance. We recommend skaters capitalize on the performance benefits of lower air resistance at higher altitudes. Additionally, adjusting technical and tactical movements based on ice surface temperature conditions enhances adaptability. Finally, heightened monitoring of fitness, fatigue, and recovery is crucial throughout the season. To conclude, our research enhances understanding of performance mechanisms and offers substantial guidance for speed skaters in optimizing responses to external factors for peak competitive performance.

## Data Availability

The original contributions presented in the study are included in the article/Supplementary Material, further inquiries can be directed to the corresponding author.
